# Meaning in Life, Future Orientation and Support for Violent Radicalization Among Canadian College Students During the COVID-19 Pandemic

**DOI:** 10.3389/fpsyt.2022.765908

**Published:** 2022-02-11

**Authors:** Diana Miconi, Gabrielle Geenen, Rochelle L. Frounfelker, Anna Levinsson, Cécile Rousseau

**Affiliations:** ^1^Department of Psychopedagogy and Andragogy, Université de Montréal, Montréal, QC, Canada; ^2^Department of Epidemiology, Biostatistics, and Occupational Health, McGill University, Montréal, QC, Canada; ^3^Division of Social and Cultural Psychiatry, McGill University, Montréal, QC, Canada

**Keywords:** violent radicalization, college students, meaning in life, future orientation, prevention, positive youth development

## Abstract

The COVID-19 pandemic has increased levels of uncertainty and social polarization in our societies, compromising young people's capacity to envision a positive future and maintain a meaningful sense of purpose in life. Within a positive youth development framework, the present study investigates the associations of a positive future orientation, presence of and search for meaning in life, and support for violent radicalization (VR) in a diverse sample of Canadian college students. In addition, we investigate the moderating role of future orientation in the association between presence of and search for a meaning in life and support for VR. A total of 3,100 college students in Québec (Canada) (69% female; *M*_age_ = 18.57, *SD*_*age*_ = 1.76) completed an online survey during the second wave of the COVID-19 pandemic. Results from linear mixed-effects models indicate that a positive future orientation and a higher presence of a meaning in life were negatively and independently associated with support for VR. Search for meaning in life was not associated with support for VR. The magnitude of the negative association between presence of a meaning in life and support for VR was greater among students with a more positive future orientation. Schools and colleges are in a privileged position to implement preventive interventions to support a positive future orientation and the presence of a meaning in life among young people during these challenging and uncertain times and reduce the risk of violence related to extreme ideologies in our rapidly changing society.

## Introduction

During the COVID-19 pandemic, fear and uncertainty have dominated the social landscape and jeopardized the capacity of young people to envision a positive future and maintain purpose in their lives (1, 2). Mounting evidence points to the negative consequences of the pandemic on adolescent and young adults' mental health (3–8). Simultaneously there has been an upsurge in all forms of violence associated with despair, further polarizing society (9, 10). A recent Canadian study indicates that in 2017 students aged 16–18 were at higher risk of supporting violent radicalization (VR), compared to students aged 19–21 who were at higher risk in 2015 (11). This age trend suggests that societal challenges over time may render younger people more vulnerable to VR. It is therefore essential that we invest in ways to support youth to reduce violence in society. This is particularly urgent in the present context of uncertainty and life disruption caused by the COVID-19 pandemic. In a positive youth development (PYD) perspective, the present study investigates how a youth's positive identity, in terms of positive future orientation and presence of and search for a meaning in life, is associated with support for VR in a sample of Canadian college students during the COVID-19 pandemic.

Violent radicalization (VR) is a complex and multidimensional phenomenon (12) defined as a process whereby an individual or a group increases support for violence as a legitimate means to reach a specific (e.g., political, social, and religious) goal (13). Although the association between support for VR and violent action is not linear, population-wide attitudes toward legitimizing some forms of violence may increase social polarization and fuel the emergence of extremist groups, thus providing a narrative to channel despair and rage in vulnerable individuals (14, 15). Schools and colleges are important radicalization vectors and recruitment sites (16). Data show that 75% of lone actors in North America and Europe received post-secondary education (17). The present study focuses on late adolescents and early adults (aged 16–25) attending colleges in Québec (Canada). In Québec, colleges (known as Cégeps) are a public educational institution placed between high school and university. Their purpose is to assist in continuing education. These types of schools provide 2–3-year preuniversity programs and vocational career programs to younger students (starting from age 16) as well as older professionals; roughly 70% of students attend college directly after high school in Quebec (18). Schools and colleges are a promising location for violence primary prevention programs, as they can reach a large portion of youth (19). Unfortunately, empirical evidence on best practices and guidelines on how to support students and prevent VR are limited (20). A better understanding of risk and protective factors associated with support for VR among adolescents and young adults is crucial to inform the development of prevention programs in educational settings.

Research on support for VR among young people may benefit from the adoption of a Positive Youth Development (PYD) framework aimed at fostering youth strengths across multiple domains of functioning (21). This framework postulates that the alignment of strengths and resources across contexts and developmental stages is the key for successful adaptation and transition to adulthood (22–25). PYD proposes that young people are not passive in the contexts that are likely to affect their development but active participants and is based on the belief that all adolescents have the potential for thriving if their developmental assets are nurtured. PYD promotes a public health approach to the study of VR, focusing on a resilience-based perspective to healthy development as a core component for preventive programs and policies (26). Focusing on the promotion of different paths of positive development may shed light on new non-violent ways for youth to contribute and develop a sense of belonging to their society. In addition, the promotion of PYD paths would engage youth in personal and social transformation, reducing feelings of despair and consequently disengaging them from VR processes. In line with a socio-ecological perspective, this framework acknowledges that youth-specific assets and person-context relations promoting a positive development vary across socio–cultural settings and historical times. Indeed, our global world is changing rapidly, with many societies characterized by increased violence, growing xenophobic sentiments, and socio-cultural inequalities and uncertainty, as highlighted by the COVID-19 pandemic (27–29). Prior evidence shows that self-uncertainty can motivate enhanced conviction on socially important issues and increase the appeal of extreme groups who offer a way to restore one's self-assurance and self-respect (30). In the present challenging global context, it is important to identify how to align youth strengths and their ecological contexts to promote their thriving through institutional democratic systems. In the PYD perspective, one developmental asset that may be timely and important to investigate in the present context of uncertainty and social polarization is youth's positive identity, defined as youth's ability to have a positive and optimistic vision of the future and to report a sense of meaning and purpose in life (25, 31, 32). Positive identity in youth may represent a form of empowerment, leading to reduction in feelings of despair, engagement in positive action for personal and social transformation, and ultimately prevent involvement in VR processes.

A positive future orientation and the presence of a sense of purpose in life are crucial to human wellbeing and become especially important during late adolescence and early adulthood, when youth are exploring life and career options (33–35). A positive future orientation refers to the extent of one's positive attitudes toward the future (36) across different life domains (e.g., personal, community, and world). A growing body of literature suggests that youth who report a negative future orientation are at greater risk of engaging in violence and supporting VR than youth who are more positively oriented to the future (36–39). A positive future orientation fosters hope and represents an important asset during an age of growing insecurities and anxieties, especially for late adolescent and early adults, thus contributing to a reduction of support for VR (38). Presence of meaning in life, defined as “the sense made of, and significance felt regarding, the nature of one's being and existence” (p. 81) (40) has been associated with wellbeing (41, 42) and fewer problematic health behaviors (43). However, it has yet to be investigated in association with support for VR.

The *presence* of a meaning in life is accompanied by the *search* for meaning in life, defined as the “strength, intensity, and activity of people's desire and efforts to establish and/or augment their understanding of the meaning, significance, and purpose of their lives” (p. 200) (44). In the North American context, higher search for meaning has been associated with lower wellbeing, especially at an older age, when the search and exploration of identities and purposes in life are less normative (44). However, findings on the association between search for meaning in life and wellbeing are mixed, suggesting socio-cultural and contextual variations (45, 46). For instance, in their cross-national study Lin and Chan (46) found that the search for meaning in life in adverse circumstances appears to be more useful than in benign conditions, in support of the idea that a deficiency search is functional in that it can contribute to an improvement of one's life conditions. The authors reported that search for meaning in life and wellbeing were not significantly associated in less collectivistic and more peaceful societies (e.g., Sweden, Australia). Under very uncertain circumstances, radical discourses may constitute a tempting response for youth in search of meaning (30, 47). Although this may lead in some cases to the legitimation of violence, in other situations it may lead to civic engagement and social advocacy (26, 48), two important means by which adolescents can contribute non-violently to their development and society (23). The COVID-19 pandemic represents a significant adverse event and hardship for young people which is likely to elicit a quest for meaning (49), but the role of a search for a meaning in life on young people's support for VR during the pandemic has yet to be investigated.

The presence of a meaning in life in the current social context can protect from feelings of despair and hopelessness (50). Simultaneously, a positive vision of the future reveals an optimistic perception of life and may be associated with the presence of meaning in life (50, 51). The two constructs may interact to contribute to a better understanding of what factors are protective against VR processes in young people during these challenging times (50). In addition, the role of search for a meaning in life as risk or protective factor for VR may also depend on their vision of the future, in that a positive vision of the future may support a constructive search for meaning in life, thus reducing the risks of involvement in VR processes.

In light of mounting evidence on the positive association between depression and support for VR (52–56) as well as the increase in depressive symptoms in youth during the pandemic (3, 4), depressive symptoms are an important and timely variable to consider. Depression involves a lack of hope and pessimism that might make extremist ideologies that promote agency and empowerment especially appealing and is associated with a negative vision of the future and lack of purpose in life (57). Although the three constructs are associated in depressive states, future orientation and meaning in life describe existential states stemming from a social and cultural environment. These are distinct from depression, which refers to a more generalized pessimism and negativity based on erroneous negative emotional interpretations.

The present study aims to: (1) examine the association of a positive future orientation, presence of and search for a meaning in life with support for VR in a sample of Canadian college students during the COVID-19 pandemic; and (2) investigate the moderating role of future orientation in the association between presence of and search for a meaning in life and support for VR – controlling for severity of symptoms of depression. We expect a positive future orientation and a higher presence of a life meaning to be negatively associated with support for VR. Given the scarce and mixed findings on the role played by search for a meaning in life on support for VR, two alternative hypotheses will be tested. The first hypothesis theorizes search for a meaning in life as a risk factor for support for VR and is based on findings on the negative impact that search for a meaning in life has on wellbeing in the North-American context (44). Alternatively, search for a meaning in life could be a protective factor for VR and this would support findings that documented the protective role that search for a meaning in life can have among young people under challenging and uncertain circumstances (e.g., COVID-19 pandemic) (46). We also expect the associations between presence and search of a meaning in life with support for VR to vary depending on levels of future orientation, with students reporting lower support for VR at higher levels of reported presence of a meaning in life, especially in the presence of a more positive future orientation (50). We expect a more positive future orientation to mitigate or strengthen the association between search for a meaning in life and support for VR, depending on whether search for a meaning in life is a risk or a protective factor for support for VR.

## Materials and Methods

### Participants

A total of 3,100 participants aged between 16 and 25 years (*M*_*age*_ = 18.6; *SD*_*age*_ = 1.76) were recruited across 18 colleges located in different areas of Quebec, Canada. Participants‘ socio-demographic characteristics are presented in Table 1. Of the 3,100 students that completed the study, 68.0% (*n* = 2,107) identified as women, 27.5% (*n* = 852) as men, and 2.5% (*n* = 79) as transgender or gender-diverse (TGD). An additional 2.0% of participants (*n* = 62) chose not to report their gender. Most students were non-immigrants (i.e., born in Canada from Canadian-born parents). First-generation and second-generation immigrants had varied ethnic backgrounds: 35.0% (*n* = 163) came from Europe, 16.75% from Asia (*n* = 78), 12% (*n* = 56) from South America, 9.66 % (*n* = 45) from North America, 8.80% (*n* = 41) from Northern Africa/Maghreb, 8.15% (*n* = 38) from Sub-Saharan Africa, 8.37% (*n* = 39), 5.15% (*n* = 24) from the Middle East, and 4.51% from the Caribbean (*n* = 21). Twenty-three students did not report their birthplace. Given the high heterogeneity of the students and of the regional groupings in the analyses, we controlled for generational status rather than for birthplace, which were significantly correlated (*r* = 0.26, *p* < 0.001). Most students reported having no religion (59.7%, *n* = 1,851). A substantial minority practiced Christianity (30.0%, *n* = 929), and a small minority practiced Islam (4.0%, *n* = 124) or another religion (5.7%, *n* = 175). Twenty-one people did not report their religious beliefs. About half of students reported never having financial difficulties in their household growing up (52.7%, *n* = 1,633), 33.7% (*n* = 1,045) reported sometimes having financial difficulties, and 13.4% (*n* = 414) reported often having financial difficulties. Eight people did not report this information. A total of 57.1% of students reported French as their primary language (*n* = 1,770), 14.7% reported English (*n* = 457), and 27.0% (*n* = 836) reported both English and French. Thirty-seven people did not report their primary language.

**Table 1:** Socio-demographic characteristics of participants and descriptive statistics of study variables for the total sample (*n* = 3,100).

**Characteristics**	**Total sample**			
	**(*****n*** **= 3,100)**			
	***n***		***(%)***	
**Gender**	3,038			
Woman	2,107		67.97	
Man	852		27.48	
Transgender and gender-diverse	79		2.55	
Missing	62		2.00	
**Religion**	3,079			
None	1,851		59.71	
Christianity	929		29.97	
Islam	124		4.00	
Other	175		5.65	
Missing	21		0.68	
**Generation**	3,058			
≥Third generation	2,109		68.03	
First generation	466		15.03	
Second generation	483		15.58	
Missing	42		1.35	
**Financial difficulty**	3,092			
Never	1633		52.68	
Sometimes	1,045		33.71	
Often	414		13.35	
Missing	8		0.26	
**Primary language**	3063			
French	1,770		57.10	
English	457		14.74	
Both	836		26.97	
Missing	37		1.19	
**Cégep**	3,077			
1	89		2.87	
2	41		1.32	
3	110		3.55	
4	140		4.52	
5	166		5.35	
6	49		1.58	
7	80		2.58	
8	383		12.35	
9	53		1.71	
10	233		7.52	
11	43		1.39	
12	255		8.23	
13	225		7.26	
14	15		0.48	
15	599		19.32	
16	255		8.23	
17	123		3.97	
18	218		7.03	
Missing	23		0.74	
**Depression cut-off**	2,978			
Below clinical cut-off	1,132		36.52	
Above clinical cut-off	1,846		59.55	
Missing	122		3.94	
	***M***	***SD***	**Missing (** ***n*** **)**	**Missing (%)**
Age	18.57	1.76	0	0.00
Search for meaning in life	22.66	7.55	25	0.80
Presence of meaning in life	21.68	7.37	18	0.60
Future orientation	13.28	4.21	39	0.01
Depression	2.10	0.70	122	3.90
RIS	11.62	6.23	47	1.50

### Procedure

Data were collected from January to April 2021, during the second wave of the COVID-19 pandemic in Québec. Participants were recruited by establishing partnerships with colleges across Quebec (Canada). The project was described as a research study on adaptation to the current social context in Quebec. Inclusion criteria were: being registered as a full-time student in one of the participating colleges and being between the ages of 16–25 years old. Students participated by completing an online questionnaire that was uploaded on the intranet portal of each college and remained online for a month. Participants could decide whether to complete the questionnaire in French or English and were informed that their involvement was voluntary and that responses would be confidential. Response rates varied greatly across colleges, ranging from 2 to 19%. The research ethic board of each institution gave ethics approval prior to data collection. The study protocol and procedures were approved by the Ethics Committee of the Center Intégré Universitaire de Santé et de Services Sociaux du Center-Ouest-de-l'Île-de-Montréal (CIUSSS-CODIM).

### Measures

#### Independent Variables

##### Future Orientation

Students' future orientation was evaluated by three items adapted from Saigh's Children's Future Orientation Scale (CFOS) (58). This scale consists of 16 items rated on a seven-point Likert scale (1 = not at all, to 7 = very much). It is designed to measure youths' future orientation in four subscales related to four specific life domains (i.e., work, family, social, and general). Subscale scores are averaged to derive a total composite score. For the purpose of this study, a short version of the scale including three items was administered, assessing the vision of the future of the world, of the community and of the self (i.e., I feel that the future offers many possibilities to my community). A global score was obtained by averaging the three items. Higher total raw scores indicate more positive attitudes about the future overall. Adequate psychometric properties were reported (58, 59). In this study, internal consistency (Cronbach Alpha and McDonald's Omega) (60) of the global vision of the future was 0.78 for both α and Omega.

##### Presence of and Search for a Meaning in Life

The Meaning in Life Questionnaire (MLQ) (40) consists of five items that measure the degree to which individuals feel that their life is full of meaning (Presence subscale) and five items that reflect individuals' motivation and desire to find or deepen the meaning in their lives (Search subscale). Items are scored on a five-point Likert-type scale that range from 1 (almost never) to 5 (almost always). The MLQ has been used with both adolescent and adult samples and has shown good reliability, validity, and a stable factor structure (40, 44). In our sample, Cronbach's Alpha and McDonald's Omega for the presence of meaning in life scale were both 0.89. For the search for a meaning in life scale, Cronbach's alpha and McDonald's Omega were 0.80 and 0.89, respectively.

#### Dependent Variable

##### Support for Violent Radicalization

The Radicalism Intention Scale (RIS) is a four-item subscale of the Activism and Radicalism Intention Scales (ARIS) (61). It assesses an individual's readiness to participate in illegal and violent behavior in the name of one's group or organization. Respondents rate their agreement with four statements on a seven-point Likert scale, with higher scores indicating more support for VR (range 4–28). A sample item is: “I would continue to support an organization that fights for my group's political and legal rights even if the organization sometimes resorts to violence.” The scale has good psychometric properties among young adults (62, 63). Cronbach's Alpha and McDonald's Omega for the sample were both 0.85.

#### Covariates

##### Depression

Depression was measured by using the 15-item scale of the Hopkins Symptom Checklist-25 (HSCL-25) (64). Items are rated on a Likert scale from 1 (not at all) to 4 (extremely), and a total score is obtained by computing the mean of all items. The clinical cut-off is set at 1.75 (score range from 1 to 4). The HSCL-25's psychometric qualities have been well-established (65). In this study, Cronbach's alpha and McDonald's Omega for the depression score were both 0.92. Depression is included in the analyses as a potential confounder in the relationship between the independent variables and support for VR.

##### Socio-Demographic Variables

Participants answered specific questions on their socio-demographic background, providing information on their age, gender (i.e., man, woman, transgender, and gender-diverse), religion beliefs (i.e., non-religious, Christian, Muslim, and Other), generational status (i.e., first-generation immigrant, second-generation immigrant, and third generation immigrant/non-immigrant), language (i.e., French, English, Both), financial difficulties (yes/no), and college. All socio-demographic variables are included and controlled for in the analyses because they are potential confounders in the relationship between the independent variables and outcome.

### Data Analysis

Analyses were performed using *R* software (66). Descriptive information for the sample was summarized using means and standard deviations for continuous variables and counts and proportions for categorical variables. We used ANOVA and Chi-squared tests to examine differences in future orientation, presence of and search for a meaning in life and support for VR according to students' socio-demographic characteristics.

Missing data for each study variable are reported in Table 1. Missing values for both continuous and categorical variables were imputed using multiple imputations by chained equations (*n* = 5) (67). Sensitivity analysis indicated that missing data and multiple imputations did not alter the observed patterns of associations.

We used linear mixed-effects models to test the separate contributions of future orientation, presence of and search for a meaning in life to support for VR (three models, one per each predictor), controlling for socio-demographic variables (i.e., gender, age, immigrant status, religion belief, financial difficulties, language) and depression, while accounting for the clustered nature of the data (i.e., students nested within colleges). Students from the same institution are expected to respond more similarly than students from different institutions as there are other institutional factors that can impact the response. Therefore, our statistical analysis accounts for this intra-institution correlation by using multi-level regression analyses. Specifically, a series of linear mixed-effects models were performed using lme4 package in R (68). Prior to all analyses, scores of support for VR, future orientation, presence of and search for a meaning in life were standardized to a mean of 0 and a standard deviation (SD) of 1.

To investigate the independent associations of future orientation, presence of and search for a meaning in life with support for VR, we implemented a linear mixed-effects model with support for VR as the dependent outcome and future orientation, presence of and search for a meaning in life as independent variables, controlling for depression, gender, age, immigrant status, religion, financial difficulties and language as fixed effects and colleges as a random effect.

Last, we conducted moderation analyses. Future orientation was categorized into quintiles to simplify the interpretation of results from effect modification analyses and allow for a stratified analysis by levels of future orientation. We ran two separate models. In the first model, we included a two-way interaction between presence of meaning in life and future orientation, controlling for the same covariates and search for a meaning in life to explore potential effect modifications. Subsequently, we tested the two-way interaction between future orientation and search for a meaning in life, controlling for covariates and presence of a meaning in life.

## Results

At the bivariate level, Pearson correlations among our variables of interest indicated that search for a meaning in life and depression were both positively associated with support for VR, whereas future orientation and presence of a meaning in life were negatively associated with support for VR as well as with depression. Search for a meaning in life was positively associated with depression, and negatively associated with presence of a meaning in life and future orientation. Presence of a meaning in life was positively associated with future orientation. In terms of age, older students reported higher presence of meaning in life but a lower future orientation (Table 2). The association of socio-demographic variables with our variables of interest are reported in Table 3.

**Table 2:** Pearson correlation matrix for continuous predictors.

***Variable***	***1***	***2***	***3***	***4***	***5***	***6***
**College participants (*****n*** **= 3,100)**						
1 Age	–	−0.01	0.05^**^	−0.06^**^	0.02	−0.04
2 Search for meaning		–	−0.34^***^	−0.13^***^	0.33^***^	0.11^***^
3 Presence of meaning			–	0.48^***^	−0.44^***^	−0.18^***^
4 Future orientation				–	−0.42^***^	−0.18^***^
5 Depression					–	0.18^***^
6 RIS						–

** *p < 0.01*;

*** *p < 0.001*.

**Table 3:** Socio-demographic characteristics of participants and descriptive statistics of study variables, separately for each socio-demographic category.

**Characteristics**	**Search for meaning in life**			**Presence of meaning in life**			**Future orientation**			**RIS**		
	***n***	***Mean (SD)***	***p***	***n***	***Mean (SD)***	***p***	***n***	***Mean (SD)***	***p***	***n***	***Mean (SD)***	***p***
**Gender**	3,014		<0.001	3,020		<0.001	2,999		<0.001	2,991		<0.001
Woman	2,092	23.06 (7.25)		2,093	21.74 (7.14)		2,078	13.43 (4.08)		2,073	11.44 (5.96)	
Man	843	21.58 (8.05)		848	22.00 (7.69)		842	13.25 (4.38)		839	11.31 (6.28)	
Transgender and gender-diverse	79	23.49 (8.13)		79	18.30 (8.53)		79	10.76 (4.59)		79	18.11 (7.36)	
**Generation**	3,033		<0.001	3,040		0.283	3,019		<0.001	3,011		<0.001
≥3rd generation	2,092	22.24 (7.49)		2,101	21.62 (7.23)		2,085	13.53 (4.16)		2,082	11.29 (6.09)	
1^st^ generation	462	23.40 (7.72)		460	22.10 (7.60)		457	12.64 (4.43)		454	11.55 (6.35)	
2nd generation	479	23.84 (7.56)		479	21.36 (7.70)		477	12.69 (4.11)		475	13.11 (6.49)	
**Religion**	3,055		0.051	3,061		<0.001	3,040		<0.001	3,033		<0.001
Non-religious	1,835	22.65 (7.54)		1,840	20.71 (7.27)		1,831	13.09 (4.14)		1,831	12.06 (6.30)	
Christianity	922	22.58 (7.50)		923	23.27 (7.08)		916	14.02 (4.16)		908	10.37 (5.76)	
Islam	123	21.24 (8.77)		123	24.82 (8.07)		118	11.91 (4.64)		119	11.13 (6.16)	
Other	175	23.71 (6.86)		175	21.54 (7.19)		175	11.14 (4.66)		175	13.51 (6.74)	
**Financial difficulty**			<0.001	3,074		<0.001	3,053		<0.001	3,045		<0.001
Never	1,627	21.95 (7.37)		1,623	22.26 (7.13)		1,633	13.94 (4.01)		1,612	11.18 (5.94)	
Sometimes	1,031	23.26 (7.59)		1,039	21.28 (7.37)		1,045	12.83 (4.21)		1,027	11.94 (6.33)	
Often	409	23.93 (7.89)		412	20.48 (8.02)		414	11.79 (4.49)		406	12.52 (6.95)	
**Primary language**	3,038		<0.001	3,045		0.032	3,024		<0.001	3,016		<0.001
French	1,754	21.99 (7.38)		1,761	21.97 (7.14)		1,745	13.62 (4.04)		1,744	10.94 (5.85)	
English	455	24.69 (7.49)		454	21.14 (7.53)		452	12.87 (4.23)		445	13.20 (6.77)	
Both	829	23.01 (7.76)		830	21.35 (7.71)		827	12.80 (4.46)		827	12.17 (6.50)	

*P-value of the effect of each socio-demographic variable on each study variable is reported*.

### Association of a Positive Future Orientation, Presence of and Search for a Meaning in Life With Support for VR

Transgender and gender diverse students reported higher support for VR compared to students who identified as women. Second-generation immigrant students reported greater support for VR compared to third-generation immigrant and non-immigrant students. Older students, religious students and francophone students had lower levels of support for VR compared to those who were younger, did not identify as having a religious affiliation, and were anglophone. Financial difficulties were not associated with support for VR at the multivariable level. After adjusting for potential confounders (i.e., gender, age, religion, generation, financial difficulties, primary language and depression, and after accounting for variability across colleges), future orientation was significantly and negatively related to support for VR. A 1-*SD* increase in future orientation was associated with a 0.10 *SD* decrease in support for VR (χ2 (df) = 24.63(1), *p* < 0.001; β = −0.10; *SE* = 0.02, *p* < 0.001). This effect persisted once controlling for presence and search for meaning in life (β = −0.07, *SE* = 0.02, *p* = 0.001; see Table 4).

**Table 4:** Results from linear mixed-effects model with support for violent radicalization as dependent variable.

**Variable**	**β**	**SE**	***p***	**χ^2^**	***p*(χ^2^)**
Gender				63.72	<0.001
Man	<0.01	0.04	0.923		
Transgender and gender diverse	0.85	0.11	<0.001		
Age (16–25)	−0.03	0.01	0.006	7.61	0.006
Immigrant status				16.63	<0.001
First generation	0.03	0.05	0.531		
Second generation	0.20	0.05	<0.001		
Religion				37.08	<0.001
Christianity	−0.21	0.04	<0.001		
Islam	−0.24	0.10	0.016		
Other	0.09	0.08	0.262		
Financial difficulty				3.03	0.220
Sometimes	0.06	0.04	0.138		
Often	0.07	0.06	0.186		
Primary language				13.65	0.001
English	0.22	0.07	0.001		
French and English	0.10	0.04	0.013		
Depression	0.07	0.02	0.002	11.19	<0.001
Future orientation	−0.07	0.02	0.001	12.33	<0.001
Presence of a meaning in life	−0.06	0.02	0.004	8.83	0.003
Search for a meaning in life	0.03	0.02	0.151	2.19	0.139

*SE, standard error. Baseline category for gender was “woman.” Baseline category for immigrant status was “third generation immigrant/non-immigrant.” Baseline category for religion was “non-religious.” Baseline category for primary language was “French.” Baseline category for financial difficulty was “never.” College was included as a random effect in the model*.

Presence of meaning in life was significantly and negatively associated with support for VR after adjusting for potential confounders. A 1-*SD* increase in presence of meaning in life was associated with a 0.10 *SD* decrease in support for VR (χ2 (df) = 25.36(1), *p* < 0.001; β = −0.10; *SE* = 0.02, *p* < 0.001). This effect persisted when controlling for future orientation and search for meaning in life (β = −0.06, *SE* = 0.02, *p* = 0.004; see Table 4).

With regards to search for a meaning in life, for 1-*SD* increase in search for meaning in life, there was a 0.04 SD increase in support for VR (χ2 (df) = 5.08 (1), *p* = 0.024; β = 0.04; S*E* = 0.02, *p* = 0.029). However, after controlling for future orientation and presence of meaning in life, the association between search for a meaning in life and support for VR was not statistically significant (see Table 4).

### Moderating Role of Future Orientation in the Association Between Presence of and Search for a Meaning in Life and Support for VR

**Figure 1:**
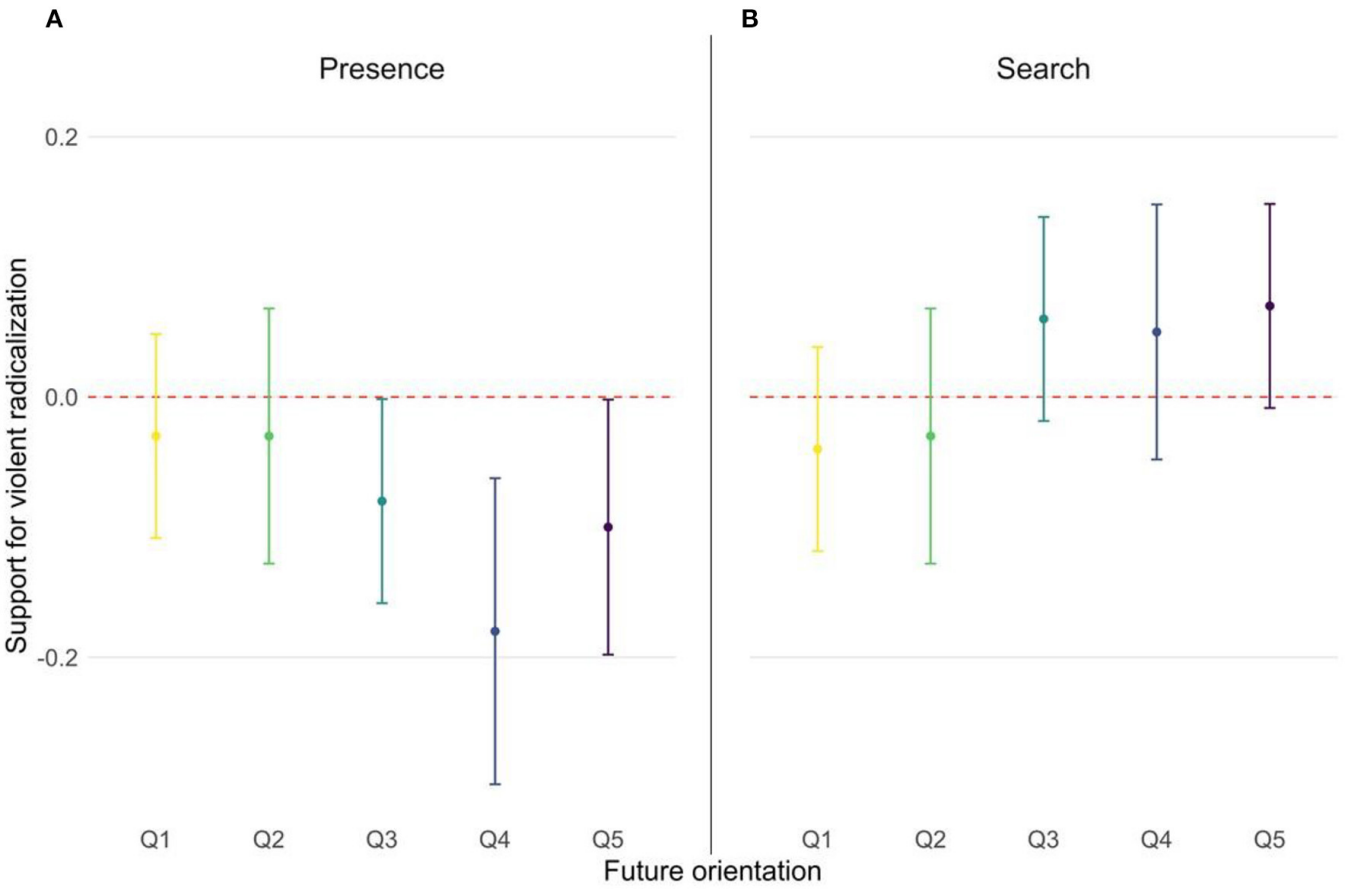
Associations between presence of meaning in life **(A)** and search for meaning in life **(B)** and support for violent radicalization, stratified by levels of future orientation, in the overall sample (*n* = 3,100). All models presented included relevant sociodemographic variables as covariates. Q1 represents low future orientation scores. See the online article for the color version of this figure.

The association between presence of a meaning in life and support for VR was stronger at higher levels of future orientation. Specifically, presence of a meaning in life was significantly associated with support for VR in students who reported a future orientation score in the fourth quantile (see Table 5). Specifically, 1-SD increase in presence of a meaning in life was associated with decreases in RIS scores of 0.18 (β = −0.18, *SE* =0.06, *p* = 0.003). A similar trend can be observed for future orientation scores in the third and fifth quintiles (see Figure 1), although these associations were borderline significant (*p* = 0.05) (Table 5).

**Table 5:** Associations between presence of meaning and search for a meaning in life and support for violent radicalization, stratified by levels of future orientation in the overall sample (*n* = 3,100).

**Independent variable**	**Moderator: future orientation**	**β**	**SE**	***p***
Presence of a	First quantile	−0.03	0.04	0.470
meaning in life	Second quantile	−0.03	0.05	0.543
	Third quantile	−0.08	0.04	0.051
	Fourth quantile	−0.18	0.06	0.003
	Fifth quantile	−0.10	0.05	0.058
Search for a	First quantile	−0.04	0.04	0.299
meaning in life	Second quantile	−0.03	0.05	0.511
	Third quantile	0.06	0.04	0.078
	Fourth quantile	0.05	0.05	0.270
	Fifth quantile	0.07	0.04	0.050

*SE, standard error. Two separate models were tested. All models presented included relevant socio-demographic variables and depression scores as covariates, as well presence of or search for meaning in life*.

The association between search for meaning in life and support for VR did not vary across levels of future orientation (Table 5). Although the effect estimates are low and borderline statistically significant, a trend toward search for a meaning in life being a risk factor, rather than a protective factor, for support for VR especially at higher levels of future orientation can be observed (*p* = 0.05).

## Discussion

The present study aimed to examine the association of one's positive identity (i.e., future orientation, presence, and search of meaning in life) with support for VR in a sample of college students in Quebec (Canada) during the COVID-19 pandemic in a PYD perspective. The pandemic is responsible of high levels of uncertainty and life restrictions (e.g., lockdown, social distancing) which have been associated with increasing feelings of helplessness and hoplessness as well as with increased psychological distress worldwide, especially among young people (1, 2). In this context, restoring a meaning in life and positive vision of the future may represent two promising ways to support young people and help them face the challenging uncertainty and losses brought about by the pandemic (69). Aligned with prior research (38), we found that a positive future orientation was associated with lower support for VR beyond the contribution of depression. Despite the social adversity and uncertainty associated with the pandemic in the past year, future orientation is confirmed in our study as an important protective factor that deters students from endorsing positive attitudes toward VR. In line with our hypothesis, higher presence of a meaning in life was also associated with lower support for VR beyond reported levels of psychological distress. Of importance, future orientation and presence of a meaning in life remained significantly associated with support for VR after including both in a model along with search for a meaning in life, suggesting that they have significant independent effects on VR.

In addition, results from our interaction analysis suggest a combined effect of future orientation and presence of a meaning in life on support for VR, as we found a stronger protective association between higher presence of a meaning in life and support for VR among students who reported an average-to-high level of future orientation. Our results confirm that a positive vision of the future and sense of purpose in life represent a form of empowerment for young people that can reduce feelings of despair and prevent support for VR even in times of social crisis, during a pandemic that may have significantly eroded or changed one's life plans and purpose (26, 38, 50, 69).

Correlation analysis in our sample indicated that both a positive future orientation and higher presence of a meaning in life were associated with lower levels of depression, in line with prior evidence (50, 51). Given that 62% of students in our sample scored above the clinical cut-off to our measure of depression (i.e., HSCL-25), the promotion of students' positive future orientation and sense of purpose in life represents a promising way to support young people who are presently experiencing high levels of psychological distress while at the same time contributing to the prevention of violence in our schools and societies. Of importance, scores above the clinical cut-off in the depression scale do not indicate clinical depression (70), but instead suggest important levels of distress among students, in line with growing literature before (11, 70) and during the pandemic (4, 5, 71). This literature indicates that distress is common among young people and has increased in recent years (72). This trend was observed prior to the pandemic (70, 72) and preliminary findings suggest that the pandemic has further accelerated this phenomenon of increasing mental distress among youth (73). These high levels of distress may indicate important levels of collective suffering among young people that stem from the challenges of our present times rather than from clinical conditions; as such, they should not be pathologized but instead situated and interpreted within the present social reality. Assisting students in restoring or building anew a sense of purpose and a positive vision of the future remains a promising prevention strategy under the present difficult circumstances characterized by high levels of social adversity.

It is also important to comment on the associations of socio-demographic variables and support for VR. Some of these associations confirmed prior results on college students in the Québec context, notably that non-religious and older students express less support for VR (11, 38, 52, 74). The fact that experiencing more financial difficulties is not associated with support for VR among young people confirms results from a recent meta-analysis in which socio-economic status explains only a small part of the variance of support for VR among young people (56). However, we observed some changes in the expected associations between gender, main language, immigrant generation and our outcome. Specifically, in contrast with previous research, men and women have comparable levels of support for VR (38). Transgender and gender-diverse youth emerge as the group at the highest risk of support for VR. This is in line with results of a recent survey conducted during the pandemic that highlighted high levels of support for VR as well as psychological distress among gender minorities (75). Such results point to the importance for future research to focus on this often-overlooked population who may be suffering more in the present context of social adversity. This also confirms prior studies that observed rapid shifts in gender roles in Western societies in recent years and underline the important role of gender in VR studies (76). The significantly higher levels of support for VR among anglophone and second-generation immigrant students in the present study may be due to the younger age of this sample compared to prior studies (11) as well as to the increasing social polarization in Québec related to language and immigration, as demonstrated by the ongoing polarizing debates around these issues in the province (77). Future studies are needed to further explore these associations.

Our findings on the association between search for a meaning in life and support for VR are somewhat inconclusive. Although there is some support for our hypothesis of search for a meaning in life as risk factor for support for VR, the size of the association is small and dependent on other individual characteristics. A higher search for a meaning in life was associated with higher levels of support for VR; however, this association became non-significant once presence of a meaning in life and future orientation were included in the same model. This suggests that, in accordance with other research in North America, the search for a meaning in life can represent a risk factor for VR (44). However, this association is weak and dependent on other variables such as one's future orientation and presence of a meaning in life. This result is aligned with recent findings of no association between the search for meaning and wellbeing in more individualistic and peaceful societies (46), a description that, overall, describes Canadian society. If anything, our results suggest that search for a meaning in life is weakly associated – if at all – with support for VR and that it varies considerably across contexts and individual characteristics. Such results are in line with mounting evidence on socio-cultural variations in the role played by search for a meaning in life on psycho-social adjustment and wellbeing (45, 46).

Results from interaction analysis suggest that the association between search for a meaning in life and support for VR does not vary based on level of future orientation. The observed trend toward search for a meaning in life becoming more of a risk factor for VR at high levels of future orientation needs to be further investigated before any conclusions can be drawn. It may be that a stronger search for a meaning leads to positive attitudes toward extreme and violent measures especially when the person believes in the possibility of a better future. The increase in self-uncertainty brought about by the pandemic (1, 2) may have increased the appeal of extreme groups and violent means that can offer a potential immediate way to restore one's self-assurance and life expectations (30), suddenly compromised by the economic crisis, the widening of socio-cultural inequalities, and social distancing requirements. Nonetheless, the role played by the search for a meaning in life in association with support for VR was consistently small across our analyses. Future studies should also investigate the potential moderating effect of perceived quality of life and psychological distress in the association between search for a meaning in life and support for VR, which could help to shed more light on possible differential associations.

Together, findings suggest that prevention and intervention efforts in the field of VR should focus more on promoting the presence of a meaning in life and one's future orientation. The role of search for a meaning in life in prevention and intervention efforts for VR is in need of further investigation; in light of the present findings a case-by-case evaluation of its role in prevention and intervention efforts may be needed as to support students' search and help them to find alternative non-violent ways to pursue their goals and make sense of their life.

### Limitations

There are several limitations in the present study. First, the cross-sectional design prevents us from drawing any conclusions about causality. Longitudinal studies are needed to shed light on the trajectories of associations between future orientation, presence of and search for meaning in life and support for VR. Second, we relied on an online method of recruitment that is associated with a wide variation in response rates. A selection bias of participants cannot be excluded if participation is differential. However, given the sensitivity of the topic and the pandemic context, this method of recruitment facilitated the participation of the students who would not have been comfortable in an individual interview (78). In addition, our mixed-effect models included colleges as random effects (i.e., students nested within colleges), thus allowing us to control for variability across colleges. Third, the associations between future orientation, meaning in life, depression and support for VR is likely very complex. While we have modeled depression as a potential confounder in the relationship between the independent and dependent variables, depression can also be conceptualized as mediator in the pathway between the independent variables and support for VR or effect modifier. Our cross-sectional study design limits our ability to test for depression as a mediator; our already complex model which accounts for clustering by colleges and includes future orientation as an effect moderator makes the introduction of a three-way interaction that includes depression challenging from the perspective of having the statistical power to detect relationships between variables. Longitudinal studies with larger samples are needed to explore this possibility and shed more light on the complex mechanisms involved in the associations between depression and these variables. Last, the data were collected during the second wave of the COVID-19 pandemic in Québec. Hence, results cannot be generalized to other cultural or social contexts. Given the rapidly evolving and long-lasting concerning situation of the pandemic in Québec and globally, it is important to monitor the situation over time *via* cohort and longitudinal studies.

## Conclusion

Despite these limitations, our study expands knowledge on risk and protective factors for support of VR among young people during the very difficult and stressful context of the COVID-19 pandemic. Our results provide preliminary support for the relevance of a PYD approach to the study of support of VR among young people by highlighting the important role of a positive identity for the prevention of support of VR.

Adolescents and early adults are among the age groups that have suffered the most during the pandemic in terms of mental health and social adjustment. Preliminary findings indicate that the pandemic has jeopardized young people's vision of the future and sense of purpose (1, 2). In line with a PYD perspective, we must help students realign their personal assets with their rapidly evolving social contexts, which are presently characterized by fear and uncertainty. The developmental tasks of imagining a future for oneself as a young adult and finding a purpose in life is made more complex by the COVID-19 pandemic. Educational institutions represent a suitable target for preventive interventions aimed to support a capacity to envision future and meaning in life in spite of the pervasive social uncertainties. Our findings suggest that education efforts need to consider uncertainty and rapid social changes stemming for our rapidly evolving societies and their consequences on young generations (79). Prevention programs that address youth's sense of purpose and their capacity for complex thinking in general and about the future (31, 80, 81) are promising to minimize the attraction of extremist ideologies among young people and support their wellbeing during these challenging times (82, 83). Life crafting interventions have also been proposed as potential beneficial programs to help rebuild meaning in life in response to the social suffering and loss of normalcy brought about by the pandemic, in line with positive psychology principles (69, 84). Such interventions help people set goals and make concrete plans to move toward a more positive future. PYD interventions also offer a variety of school-based program models to engage youth in positive activities that can contribute to their sense of purpose and future orientation (80). Preventive efforts should also aim to train college teachers and career guidance officers on how to support and promote a positive future orientation and a purpose in life among students who may be experiencing difficulties during this health emergency.

## Data Availability Statement

The raw data supporting the conclusions of this article will be made available by the authors upon request, without undue reservation.

## Ethics Statement

The studies involving human participants were reviewed and approved by the Ethics Committee of the Center Intégré Universitaire de Santé et de Services Sociaux du Center-Ouest-de-l'Île-de-Montréal (CIUSSS-CODIM). Written informed consent from the participants' legal guardian/next of kin was not required to participate in this study in accordance with the national legislation and the institutional requirements.

## Author Contributions

DM contributed to conception and design of the study, data analysis, interpretation of study findings, and writing the manuscript. GG contributed to data preparation and cleaning, preparation of tables, and drafting of the results. RF and AL contributed to methodological decisions and provided feedback on multiple versions of the manuscript. CR contributed to conception and design of the study, interpretation of study findings, and provided feedback on several drafts of the manuscript. The authors listed in the byline have agreed to the byline order and to submission of the manuscript in this form. All authors agreed to act as guarantor of the work.

## Funding

This work was supported by a postdoctoral fellowship from the Fonds du Recherche du Québec Société et Culture (FRQSC) to DM.

## Conflict of Interest

The authors declare that the research was conducted in the absence of any commercial or financial relationships that could be construed as a potential conflict of interest.

## Publisher's Note

All claims expressed in this article are solely those of the authors and do not necessarily represent those of their affiliated organizations, or those of the publisher, the editors and the reviewers. Any product that may be evaluated in this article, or claim that may be made by its manufacturer, is not guaranteed or endorsed by the publisher.
